# Inside the April 2023 Issue

**DOI:** 10.24908/pocus.v8i1.16481

**Published:** 2023-04-26

**Authors:** Benjamin T Galen

**Affiliations:** 1 Editor-in-Chief, POCUS Journal

**Keywords:** POCUS Journal, letter from the editor

Dear Readers,

With this April issue, we are pleased to announce that POCUS Journal has been recently indexed on PubMed, providing us with the opportunity to reach a wider audience and showcase our high-quality scholarship to the global medical community. We are thrilled to join the ranks of other top-tier journals indexed on PubMed and are committed to continuing to publish articles of the highest quality that advance POCUS in medicine. We are the only free, open-access, peer-reviewed POCUS journal in the world. 

We have the best managing editor in the business, Julia Herr, who we all owe a debt of gratitude for her efforts to bring us into the PubMed era. We are also grateful to Courtney McNamara, Barkha Sirwani, and Braeden Hill for their help in this process. Dr. Casey Glass of our Editorial Board played a large part in this process as did POCUS Journal founder and former editor-in-chief Dr. Amer Johri. We would also like to thank our publisher Cinquill and our advertisers for their support. 

The April issue of POCUS Journal hosts numerous outstanding case files/case reports as well as innovations in POCUS curriculum. We are also delighted to showcase a variety of research from around the world, a review article on diastolic dysfunction, and a research protocol. 

Please find our author guidelines here: 

https://pocusjournal.com/author-guidelines/

Sincerely,

Benjamin T. Galen, MD

Editor-In-Chief, POCUS Journal

